# Wait times for publicly funded addiction and problem gambling treatment agencies in Ontario, Canada

**DOI:** 10.1186/1472-6963-13-483

**Published:** 2013-11-19

**Authors:** Rachael V Pascoe, Brian Rush, Nooshin Khobzi Rotondi

**Affiliations:** 1 Factor-Inwentash Faculty of Social Work, University of Toronto, 246 Bloor Street W, Toronto, ON M5S 1 V4, Canada; 2 Social and Epidemiological Research Department, Centre for Addiction and Mental Health, 33 Russell Street, Toronto, ON M5S 2S1, Canada; 3 Department of Psychology, HIV Prevention Lab, Ryerson University, Jorgenson Hall, 8th Floor, 350 Victoria Street, Toronto, Ontario M5B 2 K3, Canada

**Keywords:** Drug use, Problem gambling, Wait times, Intake

## Abstract

**Background:**

This study describes the definitions of wait times and intake processes used by drug and problem gambling treatment agencies in Ontario, Canada, as well as the various strategies employed to ameliorate client backlog.

**Methods:**

An online survey was developed and distributed to 203 publicly-funded, provincial substance use and problem gambling treatment agencies from June to August, 2011. All aspects of the intake process were covered in the survey.

**Results:**

Based on 139 responses, six different wait time periods were identified. Additional analyses were completed by type of service offered. Suggestions for effective interventions to shorten wait times and recommendations for future research are provided.

**Conclusion:**

The results of this study highlight a need for standardized definitions of wait times across substance use and problem gambling treatment services.

## Background

In many countries around the world, health care systems are plagued with long wait times and therefore poor access to needed services. Wait times are particularly challenging in countries with universal health insurance, as waiting lists are used to ration or restrict access to services [1]. In Ontario, Canada’s largest province, wait times have long been a politically-charged issue. A strategy for reducing wait times was developed to improve access to key health services, such as cancer surgery, cardiac procedures, and hip and knee replacement [2]. However, the issue of wait times in addiction services is woefully understudied; despite the fact that it is recognized as a key component of performance measurement for treatment initiation and engagement [3]. In a field currently focused on the efficiency with which clients flow through the treatment system [4,5], measuring and understanding wait times has become an ever more important issue. In this paper, we explore the definitions and interpretations of wait lists across all publicly-funded substance use and problem gambling treatment agencies in Ontario, as well as strategies for managing and reducing wait times.

### Definitions of wait lists for addiction services

Wait lists have been defined as a “queue of patients who are deemed to need a health service that is in short supply relative to demand” [6] or “the function both of whether prospective patients can get into the queue and how quickly they get off the queue and into treatment” [7]. There is little discussion on developing systems for monitoring waiting lists and there seems to be no consensus on the steps or definitions used to measure wait times. Many of the examples in the literature consider the point at which the wait list period begins as the client’s first contact with the agency in question [8-10]. Other studies split the wait period into two: from the first contact to the initial assessment, and from that assessment to actual treatment [8,9]. Wait times have also been measured from initial assessment to entering treatment [11,12]. Some researchers amalgamate these two time periods, and define the waiting period as the amount of time between the point of first contact and the beginning of the treatment [13-16]. Alternatively, the wait period can be viewed as the length of time between the client’s decision to seek treatment to the time they actually present for services [10,17], or the time clients spend waiting for long term treatment while receiving short term services [18]. Thus, while there is agreement on the general definition of wait lists, there is no clear consensus as to what time period constitutes a wait time. Furthermore, no standard definition for wait times has been established for addiction treatment agencies.

### Effects of wait times on clients

The literature strongly suggests that wait lists are detrimental to clients and that, where possible, treatment on demand is the best operational model [7,19]. Longer wait times can contribute to pre-treatment attrition and low rates of treatment retention [10,20-22]. Wait times can also negatively impact client health outcomes and various socio-environmental factors, such as unhealthy drug using behaviours (e.g., syringe borrowing) and involvement in the criminal justice system [18,23]. These consequences are described below.

### Drop-out before treatment

Studies have shown that the presence of a wait list for addiction treatment is a significant determinant of pre-intake attrition [10,20-22]. According to Festinger et al., appointments that were scheduled on the same day as first contact had a much higher attendance rate than those with a lengthier wait [22]. Wait lists can also be a major deterrent for clients who are considering treatment but have not yet made first contact or received a referral; Pollini, et al. found that the most significant reason for not enrolling in treatment was the prospect of being placed on a wait list [21].

### Societal and individual-level impacts

While wait lists may influence treatment attrition, the harms related to problematic substance use may be lessened for those who receive prompt treatment. An important benefit of the treatment-on-demand model is the immediate reduction or elimination of substance use for those clients who self-refer, as they would be particularly motivated to change [9,19]. Wait-listed clients, like those receiving outpatient services, are still vulnerable to the risks of drug use during this period; however, no differences have been found in the frequency and quantity of drug use when compared to those not on a wait list [8].

Wait lists are associated with negative health and social consequences for clients. Chun, et al. compared clients that were waiting for treatment for two months to those waiting over two months [18]. The latter group experienced greater employment problems [18], possibly due to their inability to join the workforce as clients had to be available at all times if called in to treatment [24]. In another study, injection drug users reported syringe borrowing while on wait lists, thereby putting them at risk of acquiring blood borne infections through needle sharing [25]. Although there are variations in how clients cope with their drug use while on wait lists, some continue to use substances, citing a loss in motivation after being told they would have to wait for treatment [24]. Other clients may use a form of harm reduction by limiting their use to one type of drug or using another drug instead [24]. Conversely, approximately 35% of individuals with a history of substance use, but who reported no current problems at the time of initiating the treatment queue, resumed using substances while on waiting lists for treatment [26,27]. Redko found some clients reported intentionally overdosing or bingeing in order to more quickly be admitted to medical addiction services [24]. Furthermore, waiting for treatment may contribute to involvement in the criminal justice system [28]. Adamson studied clients wait listed for methadone maintenance and found they were involved in the sex-trade, committed property crimes and other drug related crimes, putting themselves and the community at risk of violence and financial loss [23].

Overall, the different operational definitions of wait times used across studies makes interpreting the literature problematic. For instance, Addenbrooke and Rathod [13] and Claus and Kindleberger [28,29] found contradictory results for rates of treatment retention compared across wait periods of different lengths. This is likely because Addenbrooke and Rathod measured wait times from the point of first contact to assessment [13], while Claus and Kindleberger used the point of assessment to treatment at a referred agency [29]. Uniform definitions for wait periods are critical for cross-study comparisons.

### Wait time interventions in the literature

Although no clear strategies in decreasing wait times emerge from the literature, various interventions have been found to reduce client attrition and increase client involvement in the treatment process. For instance, a Strengths Based Brief Solution Focused Counseling Model, which involves working on client goals while focusing on their strengths to create solutions in a short time-frame, was proposed by Mireau, et al. in order to reduce wait times. The authors found that the intervention increased the number of clients interested in the program as well as treatment completion [30]. Interestingly, in spite of the increase of clients interested in the intervention, there were shorter wait times for treatment entry [30]. It can be argued that a community-based model that emphasizes client-centered care can provide help and support at a time when clients are feeling vulnerable and lost in an over-burdened, impersonal system.

Researchers have previously explored the effects of wait lists on pre-treatment attrition and client health outcomes. Our goals are more modest but equally important in examining the broad range of definitions in wait times across a large system of treatment agencies in Ontario, Canada. Additionally, interventions to shorten wait times will be described in an effort to recommend intake processes and policies that may reduce client backlog. Understanding variation in the definition of wait times across this sector and how wait times are being managed is important for developing evidence-informed interventions, identifying system performance measurement indicators and supporting evaluation research.

## Methods

A literature search and review was undertaken in order to identify pertinent research in the area, including examples of relevant questionnaire items, using online databases PsycINFO, Embase, Ovid MEDLINE and Scholars’ Portal. Government reports and grey literature were also pulled from generic search engines and official websites. No research was found on the effects of wait lists specifically for problem gambling interventions.

Site visits to six addiction and problem gambling treatment agencies were made between June and August, 2011. The sites were selected to be representative in terms of geographic location, agency size and service type, and included large hospitals in cities, as well as community-based organizations in both urban and rural settings. Intake services, residential and day treatment programs were observed and a variety of agency staff members were consulted. During the visits, information about individual intake and referral processes, as well as wait list procedures were documented. This information was helpful in streamlining the literature review process by focusing the search on articles that specifically addressed intake processes. The information that was compiled during the site visits also informed the development of relevant questions for an online survey of all addiction agencies in the province. Furthermore, intake workers, managers and support staff at the six sites reviewed the preliminary questions and suggested revisions based on their field experiences. The questionnaire was also reviewed by ConnexOntario (http://www.connexontario.ca/), an organization that provides information about local treatment services and supports, including estimated wait times, to individuals seeking addiction, problem gambling or mental health services. Agents connect service users to available programming that is suitable to their needs [31].

Questionnaire design was mainly based on Donmall, Watson, Millar and Dunn’s Outcome of Waiting Lists (OWL) Study [32]. The OWL paper investigated wait lists of substance use treatment programs serving opiate users in England. This project included a questionnaire of the British drug services to elucidate the system factors that affect waiting lists. Of the 57 questions in our online survey, approximately 40% were informed by the OWL questionnaire. A copy of the questionnaire is provided in Additional file 1.

The final questionnaire was completed online using the website surveymonkey.com; it contained multiple sections, including Participant Information, Definitions, Priority Status, Point of First Contact, Assessment, Drop Out and Wait Times. A list of all 205 publicly-funded addiction and problem gambling treatment agencies in Ontario was obtained from ConnexOntario. Attempts to contact one agency were unsuccessful and another self-selected out of the contact list. The questionnaire was distributed to 203 agencies between November 21^st^ and December 9^th^, 2011. Of these, 139 questionnaires were completed (68% response rate). We made efforts to increase the response rate by sending reminder emails to all agencies during the study period. Our response rate is higher than that reported in a previous study (approximately 50%) examining wait times of substance use treatment programs in the United Kingdom [32]. Available data from all surveys were analyzed using a thematic content analysis approach; graphs were used to provide visual representations of the information.

Additional analysis was performed by type of program. The results were categorized according to responses to the following question “Service Type of your program according to ConnexOntario classification”. The specific program categories included: initial assessment/treatment planning; community withdrawal management; community treatment (comprised of community day/evening treatment, case management, community medical/psychiatric and community treatment); residential withdrawal management services; residential treatment (including residential medical/psychiatric treatment and residential support treatment); and addictions support within housing.

The Ontario Ministry of Health and Long-Term Care directs funding to the Local Health Integration Networks (LHINs) which administer funds to both addictions and problem gambling treatment programs in the province of Ontario [33]. According to the Problem Gambling and Responsible Gaming Strategy developed in 1996, 2% of revenue from Ontario slot machines and race tracks is dedicated to problem gambling research, prevention and treatment [34]. The funds for problem gambling treatment are allocated to both problem gambling and addiction services [34], in recognition of a high degree of concurrent gambling and substance use [35]. Most problem gambling treatment services were in fact developed within existing addiction programs when the new funding was introduced by the Problem Gambling and Responsible Gaming Strategy [34,36]. Therefore, problem gambling and addiction treatment services in Ontario are not necessarily seen as distinct and separate services; rather, the overlap between the conditions is acknowledged through the shared funding structure.

### Ethical considerations

Our study involved interaction with individuals who were not themselves the focus of the research. Data was collected from authorized personnel at publicly-funded treatment agencies in order to release information about policies, procedures and professional practices. According to the Government of Canada’s Tri-Council Policy Statement: Ethical Conduct for Research Involving Humans (see http://www.ethics.gc.ca/pdf/eng/tcps2/TCPS_2_FINAL_Web.pdf), studies including such individuals do not require ethics review and approval. As a result, written informed consent was not obtained for participation in the study. However, all individuals were informed that completing the online survey was voluntary, and that their participation would be anonymous and confidential. Completion and return of the questionnaire was considered implied consent.

## Results

**Figure 1:**
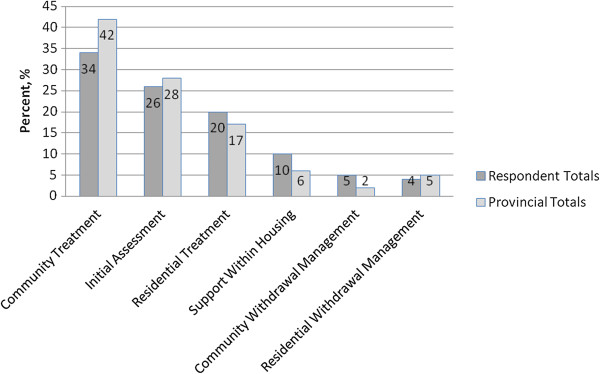
**Distribution of Programs Offered by Particiaption Offered by Participating Agnecies vs. All Publicly-Funded Treatment Agencies in Ontario, 2011.** This figure describes the distribution of respondent agencies by type of program. Respondent program information was compared to Ontario provincial programs. The respondent sample accurately represents provincial program totals.

The results include survey responses from intake workers, clinical supervisors, project managers and program, clinical and executive directors who were familiar with the intake process. The diversity of programs offered by participating agencies vs. all publicly-funded agencies in Ontario is shown in Figure 1. Overall, the distribution of programs in our sample is representative of provincial totals.

### Definitions of wait lists

The respondents were asked to define their wait periods and several different definitions were reported, including:

a. Assessment to treatment (37%).

b. First contact to assessment (19%).

c. First contact to treatment (19%).

d. Referral to admission into treatment (10%).

e. The wait between referral and the agency’s decision to admit the client into the program (8%).

f. First contact to housing or orientation (6%).

**Figure 2:**
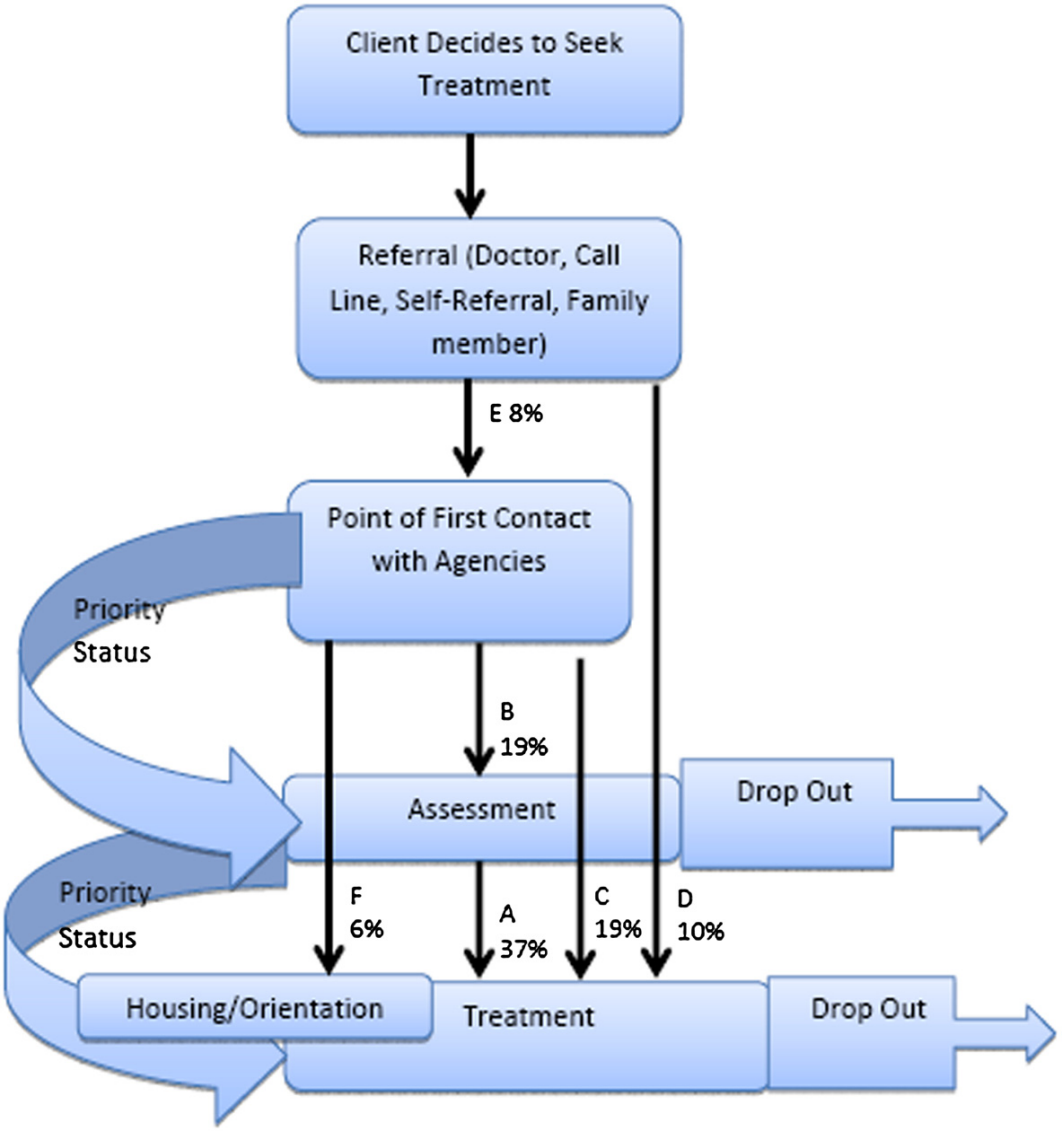
**Client Flow and Different Wait Periods in Addiction and Problem Gambling Treatment Agencies, Ontario, 2011.** This figure details the client flow through the treatment entry process. Wait periods identified by respondents are indicated with a black arrow. The percentages of responses indicating each wait period are identified.

The various wait periods are also represented in Figure 2 below.

### Wait lists

Of all respondents, 65% maintained a waiting list. This varied by program, ranging from just over half of the agencies that provided community treatment and initial assessment services to 83% for those offering residential services. Two-thirds of all respondents did not find their wait list overwhelming, although about half (56%) of the agencies with community withdrawal management programs were overburdened with their wait list. A third of respondents had between 10 and 50 clients on their wait list (32%). The amounts varied greatly based on the size of the respondent agency, with some (8%) reporting zero clients on their wait lists and others (3%) with over 200 clients. The vast majority of the respondents (92%) did not set a restriction on the capacity of their wait lists. Thirteen percent of the agencies had wait lists that varied greatly across multiple programs, thus they could not accurately provide an estimate of how many clients were on their wait lists.

Over half (59%) of the respondents reported that the number of clients on their wait lists had increased in the last five years while 25% reported the number had stayed the same; 17% observed a decrease in the number of clients on their wait lists. Agencies providing residential withdrawal management services reported the largest increase in their wait lists compared to all other programs.

Finally, most respondents (71%) were unable to determine whether their clients were on multiple wait lists, i.e., to receive services from other agencies. However, when examined by program type, agencies with community withdrawal management services were more likely to track this information (33%). Furthermore, for over half of the agencies offering a variety of programs (57%), clients were not placed on multiple wait lists within the agency.

### Priority assessment

Some clients are given priority status and can bypass wait times in order to be directly admitted into treatment. A total of 29% of the respondents formally recognized some clients as being in a priority group, 26% informally recognized priority clients, 16% recognized priority clients both formally and informally, and 27% did not recognize priority clients. Agencies providing community withdrawal management programs (83%) were most likely to recognize priority groups.

Client groups that were recognized as priority included those at risk of harming themselves (51% of surveyed respondents); pregnant women (43%); people with personal safety issues (35%) or serious mental health problems (28%); homeless individuals (28%); and those with concurrent disorders (24%). Other priority status groups included: problem gambling, withdrawal management, and those who pay for services out of pocket. Most agencies emphasized that client risk varied greatly and classification was decided on a discretionary, case-by-case basis. In contrast to the above findings, most residential treatment programs recognized women (40%) and First Nations people (34%) as priority groups. Note that most respondents cited more than one priority group; therefore the total proportion exceeds 100% (see Table 1).

**Table 1:** Priority groups in addiction and problem gambling treatment agencies, Ontario, 2011

**Priority groups**	**N (%)**
At risk of harming themselves or others	42 (51)
Pregnant women	35 (43)
People with personal safety issue (e.g. threat of partner violence)	29 (35)
Other	28 (34)
People experiencing homelessness	23 (28)
People with serious mental health problems	23 (28)
Clients with concurrent disorders	20 (24)
Transfers from particular services or systems	18 (22)
Youth	14 (17)
Clients on probation	10 (12)
Serious physical health problems	9 (11)
People of first nations, metis or inuit descent	6 (7)
Offenders referred by arrest referral	5 (6)
Families	4 (5)
HIV positive status	4 (5)
Injection drug use	3 (4)
Language barrier	3 (4)

This table identifies the client groups considered priority by survey respondents. The percentage of respondents who identified a recognized category is indicated. Individuals accessing treatment who are at risk of harming themselves or others were cited most commonly as a priority treatment group (51% of respondents).

The mean number of priority referrals reported in a month was 10.6 (SD = 16.9). The large variation likely reflects major differences in the sizes of responding agencies. About half (52%) of all respondents stated that the number of priority referrals had increased in the past five years. Agencies offering addictions-related support within housing programs reported a much higher increase in priority referrals (71%). Thirty percent of all respondents attributed the increase in priority referrals to an increase in clients; however, other reasons cited included: lower stigma for clients entering treatment, changes in policy, increase in clients with complex needs, and strategies implemented to make programs more accessible for low-income clients. Agencies with community and residential withdrawal management programs specifically attributed this change to increased oxycontin use in Ontario.

The typical length of time a priority client will wait for an assessment ranged from two days to one week; however, there was great variation. For example, some priority clients waited for as long as two to four weeks for an assessment. For treatment, the typical wait time for priority clients was around three weeks, but this could vary from five days to six weeks. Some agencies provided treatment on demand for priority clients; however, the majority of respondents (77%) did not reserve beds or spaces for priority clients. Agencies reserving beds did so for individuals in the justice system, pregnant clients, First Nations clients, homeless clients, those at risk of harming themselves, and clients with concurrent problems or problem gambling issues.

### First contact

The number of non-priority clients referred to participating agencies varied greatly, ranging from 30–200 over a one-month period. The average wait between first contact and assessment was two weeks, but some clients experienced waits as short as one to two days and others as long as seven months. Over the past five years, a third of all respondents reported an increase in wait times and a minority observed a decrease (19%). Increasing wait times were attributed to changes in policy (although these policies were not specified), and an increase in clients, particularly those with more complex needs. A system of triaging clients at first contact was cited as a reason for reduced wait times.

Sixty-percent of all respondents offered services to clients while they were waiting for screening or assessment. The services offered included program meetings, weekly drop-in support programs, and access to counsellors who were available specifically for clients on the wait list. Additionally, agencies stated that clients had access to crisis lines such as DART, a drug and alcohol helpline (part of ConnexOntario) to assist in connecting clientele with appropriate services. Agencies providing residential treatment services were least likely to offer services to clients during this wait period (47%).

### Assessments

After the point of first contact, 77% of the agencies provided formal client assessments, 12% performed informal assessments, and 13% required clients to have already completed assessments from another agency (total exceeds 100% due to rounding). The wait time between assessment and treatment varied greatly between agencies, ranging from 14 to 300 days. In the past five years, the time between assessment and treatment had mostly stayed the same for half of the survey respondents (54%), increased for 28%, and decreased for 18%. When examined by program, the majority of agencies providing residential withdrawal management and support within housing services observed no change in the past five years (70%). Generally across all programs, the amount of time between assessment and treatment remained stable. Almost all of the agencies informed clients on wait lists regarding how long they would be waiting for treatment.

### Drop out

Client attrition may occur at any time along the treatment trajectory. Across all agencies, respondents indicated that 19% of clients left intake before receiving assessment, and 20% dropped out after the assessment, but before entering formal treatment. However, these estimates varied greatly by agency, ranging between 0% and 64%.

About half (46%) of the respondents performed follow-up on clients who had dropped out of their programs. Follow-up showed that drop out was most often due to relapsed drug use or problem gambling; in some cases, the client indicated not being ready for treatment. Other reasons cited for drop out included changes in address or life circumstances (making clients unavailable for treatment) and incarceration. Additionally, long wait times for treatment were also found to be a factor for clients who had left the intake process.

### Interventions to reduce wait lists

Two thirds of the respondents had strategies to reduce or eliminate wait lists. Only 46% of agencies providing residential treatment programs reported having a strategy to shorten wait times, which included referrals to community treatment. All agencies that offered community withdrawal management services implemented strategies to shorten wait times. These included group intake, group assessment, intake on a drop-in basis, a 15-session treatment model, client flow portfolio, wait list meetings, and a triaging process for clients. A majority of all respondents (73%) reported these strategies were successful in reducing wait times. Agencies providing support within housing programs were most likely to report that their interventions of group treatment and case management successfully reduced wait times (78%). When asked about the barriers to reducing wait times for treatment, 30% of respondents cited an inadequate number of beds or space. Additional barriers included reduced funding and resources as well as a growing demand for treatment. Residential treatment programs faced issues specifically related to a lack of beds, while community programs were burdened with a lack of capacity.

### Additional comments

Comments from respondents reflected their frustration with their wait list system and the need for this topic to be further researched:

We’re frustrated with having so many clients waiting for our services because clients can die with the long wait and we would like funders to [understand] this situation…

And:

It is the worst part of my job telling a patient who is ready to change and requires medical detoxification that they will need to wait up until 8 months [between wait time to get their appointment and receiving an inpatient bed] before they can receive treatment.

Many agencies listed the lack of funds as their main barrier and were confident that with an increase in funding, their wait times would decrease. One respondent put it simply:

Capacity has not been increased to meet demand, plain and simple.

Many respondents stated that clients wanted to receive treatment-on-demand which results in frustration and burn-out for staff when they cannot meet this need. Agencies without wait lists provided examples of various interventions they found helpful in shortening waiting periods such as booking appointments at first contact, integration between mental health and addiction treatment services (for those agencies with mental health services), and regular telephone contact between the agency and client during the wait period.

## Discussion

This study has demonstrated that there are a broad range of definitions for wait lists across drug and problem gambling treatment agencies. A total of six different types of wait periods were identified from the provider survey. Many of the agencies recognized the need for a more precise definition. However, developing a standard definition is no simple task, as there are various and nuanced intake procedures across addiction and problem gambling treatment services. One intervention described in the survey was a pilot project across a number of treatment sites that hoped to standardize wait list definitions by specifying wait periods by a code number. For instance, Wait 1 comprises the period between referral and decision to admit the client into the program; while Wait 2 starts from the time a client is admitted into the service to the date they receive an assessment. Three wait periods are specified with an overall wait period (Wait 1–3) to comprise the entire timeframe. This system may be useful in providing a standard policy recommendation across all addiction and problem gambling treatment agencies in Ontario and elsewhere.

### Financial constraints

A theme expressed by the respondents throughout this survey was the financial and time constraints they experienced in implementing programs and accommodating the needs of all clients. Many of the respondents cited a recent increase in clients and the inability of the agency to meet the demand financially. One agency noted that not having a wait list for treatment makes it appear as if there is no need for the service, and may result in cuts to funding. Another respondent described supplementing staff salaries with fundraised dollars because they did not receive enough funding publicly. Fundraising was cited as a successful intervention to lower wait lists. However, this option is not available to all agencies across Ontario, because of the variation in capacity to fundraise. Added to this is the fact that fundraised dollars may be allocated to other specific uses such as space or IT, leaving little for staff.

### Increase in need

Respondents attributed their wait lists to a marked increase in people seeking addiction treatment over the past five years and a parallel decrease or hold in funding. Part of the increase can be attributed to clients with problems related to oxycontin use, especially for withdrawal management services. This reflects the alarming increase in problematic opiate use in Ontario from 2005 to 2010, which was mainly driven by prescription opioid and over-the-counter codeine use [37]. Furthermore, reductions in experiences of stigma related to seeking treatment may have also contributed to higher demand for addiction services. The increase in clients and lack of funds are important issues influencing the wait times for addiction and problem gambling treatment. A system-wide review, including a comprehensive economic analysis that takes into account supply and demand in the treatment system, may be needed to inform the development of strategies that reduce the wait times for clients and burden on staff.

### Interventions to reduce wait times

This study also examined the intake processes and strategies that are effective for reducing the length of wait for treatment. These interventions included triaging clients at intake, drop-in assessments and group intake. The results from this survey point to certain strategies that may be recommended to agencies in order to lower wait times. For example, triaging clients at intake was said to be effective in reducing clients’ waits for treatment. However, the specific processes of ‘triage’ were not provided in the survey. Some agencies indicated that it is important that priority clients are seen immediately. As such, reallocating resources to stress areas in the intake process created the greatest benefits for at-risk clients. Providing triage as well as other services in the community was also cited as a future area to explore. While requiring further evaluation, this may be a strategy that could offer relief for long wait lists.

Another intervention that reduced wait times was the use of group intake and assessment. The latter can take place at various times and involve a number of clients self-administering tools at one time with an intake worker. This is followed with one-on-one interviews led by counsellors. Scheduling specific periods of time during the week for client assessment or intake provides the community with regular and consistent opportunities for seeking treatment. Other interventions included redirecting clients to other programs with less demand at that time. Residential programs were also regularly evaluated to ensure that beds were never empty. Constant monitoring of client flow by agency staff was seen as an effective approach to lowering wait times.

These interventions are practical and successful strategies to reducing wait times. All respondents were conscious of the client experience. Furthermore, 60% of respondents provided some services to clients while they were on the wait list, including referrals to other agencies. While these referrals may ameliorate the negative effects of waiting for treatment, it might be viewed as merely redirecting or even off-loading clients to other agencies. It must be acknowledged that many agencies do not have the resources or time to develop new programs to triage clients or provide drop-in assessments and regular client flow monitoring. The development and implementation of these interventions requires funding and staff, which is the main barrier faced by addiction and problem gambling treatment agencies.

### Limitations

This study has a number of limitations, some of which were reported by respondents of the survey. Larger agencies found the questionnaire difficult to complete because they had multiple programs with varying intake processes. For many agencies, there is no way of knowing whether some clients are on multiple wait lists. Therefore, wait times may actually be shorter than perceived, and clients that have dropped off a list may have received treatment at another agency.

Additionally, this questionnaire was distributed to all publicly funded agencies, which consist of programs that vary greatly in size and clientele. Due to the large range in programs and intake procedures across addiction and problem gambling agencies, creating a questionnaire that is generalizeable to all is challenging. For this reason, it may be problematic to recommend interventions to all agencies across Ontario. In fact, some agencies indicated that a few of the questions did not accurately reflect their services, while others responded positively to the applicability of the questions and breakdown of intake procedures. Ultimately, the idiosyncrasies of the varying intake processes may prove challenging to measure using survey procedures.

Another limitation of this survey is the potential for selection bias. This study may be largely representative of agencies that have the time and resources to complete the questionnaire. It is possible that agencies with the longest wait times for services were overly burdened and thus unable to participate in a research study. Furthermore, some program categories were small (for example, 11 respondents provided residential withdrawal management services), thereby limiting the reliability of the results. Nonetheless, this level of analysis serves as a preliminary investigation into the differences in intake processes and wait times between various types of addiction and problem gambling treatment programs.

Additional analyses separating problem gambling and addiction programs were attempted, but ultimately not carried out. Eighteen of the respondents stated that their responses to the survey included their problem gambling programming in combination with addictions services. Only three of those respondents completed the survey specifically for their problem gambling services, independent of their substance use programming. Therefore, our survey results are not representative of problem gambling services that are independent of substance use services in Ontario, perhaps reflecting the shared funding structure discussed in the Methods. Furthermore, as a result of the low response rate for problem gambling-only services, comparisons between problem gambling and substance use programs were not possible. Future studies should consider examining wait times specifically for problem gambling treatment services, in order to gain a full understanding of how these services differ (if at all) from substance use-only services.

Finally, telephone helpline or internet-delivered services, such as the Ontario Problem Gambling [http://www.problemgamblinghelpline.ca/] and Drug and Alcohol [http://www.drugandalcoholhelpline.ca/] help lines, were not examined in this study. Online and telephone treatment programs have been shown to be effective for Providing services via telephone or online options may allow for cost savings and shorter wait times, while still allowing for effective treatment outcomes for people dealing with problem gambling [38] and uncomplicated substance use [39]. The exploration of online- or telephone-mediated treatment services may be an additional consideration for future research in addictions and problem gambling.

## Conclusion

This study, the first of its kind in Canada and Ontario specifically, reveals that different wait times and definitions of wait periods exist across a large network of addiction and problem gambling treatment agencies. It is clear from these findings that further study is needed in a number of areas regarding the measurement of intake processes and wait lists. Our findings will be complemented and strengthened by pilot projects that are currently underway across some agencies to standardize wait list definitions. Additional research may also help to empirically determine if certain strategies, such as triaging and group intake, are effective in reducing client backlog. However, it must be noted that agencies face important barriers to establishing intervention programs, such as a lack of funding and limited staff resources. Furthermore, this study serves as a preliminary exploration into a complex, system-level issue related to addiction and problem gambling wait times. The policy implications emerging from this study are therefore only broad recommendations; future reports should explore and evaluate more specific guidelines for addiction and problem gambling services in Ontario.

Ultimately, this research is about the client experience. Much of the literature describes client dissatisfaction with the length of wait times, and the various ways individuals cope while waiting for treatment. During the site visits at the initial phases of this project, discussions between researchers and clients revealed similar dissatisfactions with the long waits for service. One client had to wait for over a year to receive treatment and reached out to her Member of Parliament to voice her complaints. The experience of the clinicians must also be noted, as many of the responses demonstrated exasperation and frustration with long wait lists. The issue of wait times is important in improving the treatment process for clients and ensuring agencies are able to adequately meet the demands of clients. Although wait times may be measured differently across Ontario, clients are still waiting for treatment from the moment they decide to seek it to the point they engage in the intervention. This study has revealed that differences in wait list definitions exist across Ontario and that certain interventions developed by agencies are effective in reducing wait times. Future research is required to ensure wait list definitions are standardized and interventions for reducing wait times are formally evaluated in order to establish best-practices.

## Competing interests

The authors declare that they have no financial or non-financial competing interests.

## Authors’ contributions

RP carried out the literature review, survey development, implementation and analysis as well as drafted the manuscript. NKR oversaw the design of the study and contributed to the final version of the manuscript. BR conceived of the study, oversaw the design and coordination, and contributed to the final version of the manuscript. All authors read and approved of the final manuscript.

## Authors’ information

At the time of study implementation and writing, RP was an undergraduate student at the University of Toronto, in a studentship role at the Centre for Addiction and Mental Health. At the time of submission and publication, RP is a Master of Social Work student at the Factor-Inwentash Faculty of Social Work at the University of Toronto. At the time of study implementation, NKR was a project scientist at the Health Systems and Health Equity Research Group at the Centre for Addiction and Mental Health. NKR is currently a Postdoctoral fellow at Ryerson University. BR is scientist emeritus in the Social and Epidemiological Research Department at the Centre for Addiction and Mental Health.

## Pre-publication history

The pre-publication history for this paper can be accessed here:

http://www.biomedcentral.com/1472-6963/13/483/prepub

## Supplementary Material

Additional file 1: **This file contains the questionnaire, created on surveymonkey.com, and sent to addiction and problem gambling services in Ontario.** The questionnaire contains nine sections: Organization Information, Respondent Information, Definitions, Priority Status, First Point of Contact for Non-Priority Clients, Assessment, Drop Out, Wait Times, and Survey Completion. Click here for file
