# Supplementary material for: Wait times for publicly funded addiction and problem gambling treatment agencies in Ontario, Canada
Source: BMC Health Serv Res. 2013 Nov 19;13:483. doi: 10.1186/1472-6963-13-483 (PMC3874680; doi:10.1186/1472-6963-13-483)
Supplement: Additional file 1: — This file contains the questionnaire, created on surveymonkey.com, and sent to addiction and problem gambling services in Ontario. The questionnaire contains nine sections: Organization Information, Respondent Information, Definitions, Priority Status, First Point of Contact for Non-Priority Clients, Assessment, Drop Out, Wait Times, and Survey Completion. [file 1472-6963-13-483-S1.pdf]

Ontario Addiction Treatment Organizations Wait Times Study

Organization Information

Currently, little data exists on the nature of wait times for Ontario Addiction Treatment Centres. Therefore the definition of wait times may vary, making comparisons and generalizations across Ontarian organizations impossible.
The Ontario Addiction Treatment Organization Wait Times Study seeks to capture the nature of Ontario Wait Lists in a survey submitted to agencies across Ontario. By completing this questionnaire, further knowledge of definitions and nature of programs will be gleaned in an effort to ameliorate the problems many agencies and clients face while waiting for the treatment process to begin.

***1. Name of Organization:**

2. Location:

3. LHIN(s) (check all that apply):

- ☐ Erie St. Clair
- ☐ South West
- ☐ Waterloo Wellington
- ☐ Hamilton Niagara Haldimand Brant
- ☐ Central West
- ☐ Mississauga Halton
- ☐ Toronto Central
- ☐ Central
- ☐ Central East
- ☐ South East
- ☐ Champlain
- ☐ North Simcoe Muskoka
- ☐ North East
- ☐ North West

4. Program:

5. For residential treatment, what is your Ministry Funded bed capacity?

6. For residential treatment, what is your non-Ministry Funded bed capacity?

Respondent Information

***7. Your Position:**

8. Service Type of your program according to Connex Ontario classification (check all that apply):

- [ ] Community Day/Evening Treatment
- [ ] Case management
- [ ] Community medical/psychiatric treatment
- [ ] Community treatment
- [ ] Community Withdrawal Management Level 1
- [ ] Community Withdrawal Management Level 2
- [ ] Community Withdrawal Management Level 3
- [ ] Initial Assessment/Treatment Planning
- [ ] Residential Medical/Psychiatric Treatment
- [ ] Residential Supportive Treatment Level 1
- [ ] Residential Supportive Treatment Level 2
- [ ] Residential Treatment
- [ ] Residential Withdrawal Management Level 1
- [ ] Residential Withdrawal Management Level 2
- [ ] Residential Withdrawal Management Level 3
- [ ] Support within Housing

9. Is this service restricted to or specifically targeted at (check all that apply):

- ☐ Acquired Brain Injured Clients
- ☐ Cocaine Users
- ☐ Concurrent Disorders
- ☐ Early Childhood Development Clients
- ☐ Families
- ☐ First Nations, Métis and Inuit Clients
- ☐ Heroin Users
- ☐ HIV/AIDS Clients
- ☐ Inhalant users
- ☐ Lesbian, Gay, Bisexual and Transgendered Clients
- ☐ Men
- ☐ Methadone Maintenance Clients
- ☐ Older Adults
- ☐ Problem Gambling
- ☐ Women
- ☐ Youth

Other (please specify)

Definitions

10. In your own words, please describe how your organization defines the point of first contact:

11. In your own words, describe how your organization defines or provides intake:

12. In your own words, please describe how your agency defines or provides assessments:

13. In your own words, describe how your organization defines or provides treatment:

Priority Status

Priority Status may be given to a client who fits a certain criteria to bypass a wait time or to be given 'special treatment' during the waiting or treatment process.
If your agency treats some clients as priority (e.g. pregnant women, youth, etc.) and grants them the ability to bypass the line and enter treatment earlier than other clients, click yes, as wait time information regarding a priority group may differ from non-priority clients.

14. Does your organization recognize some clients as priority in terms of access?

- Yes formally
- Yes informally
- All of the above
- No

Priority Status

15. Which groups are prioritized? (check all that apply)

- [ ] At Risk of Harming Themselves or Others
- [ ] Clients on Probation
- [ ] Clients with Concurrent Disorders
- [ ] Families
- [ ] HIV Positive Status
- [ ] Injection Drug Use
- [ ] Language Barrier
- [ ] Offenders Referred By Arrest Referral
- [ ] People Experiencing Homelessness
- [ ] People of First Nations, Metis or Inuit Descent
- [ ] People With Personal Safety Issues (e.g. Threat of Partner Violence)
- [ ] Pregnant Women
- [ ] Serious Mental Health Problems
- [ ] Serious Physical Health Problems
- [ ] Transfers From Particular Services Or Systems
- [ ] Youth
- [ ] Other (please specify)

16. Please estimate the number of priority referrals your program receives in a given month:

17. In the last five years, has this number:

- ○ Increased
- ○ Decreased
- ○ Stayed the Same

Priority Status

18. What may account for this difference? (e.g. change in policy, increase in clients)

19. What is the typical length of time a client who is considered a priority will wait between first contact with your organization and assessment in days? (If your organization does not provide clients with an assessment, please indicate in the box below):

20. What is the typical length of time a client who is considered a priority will wait between assessment and treatment in days?

21. Does your service reserve beds or spaces for certain subgroups of clients (e.g. youth, mothers)?

- ◯ Yes
- ◯ No

Priority Status

22. If yes, what groups does your service reserve spaces for?

First Point of Contact for Non-Priority Clients

Those clients who are non-priority do not have any situational or personal traits that exempt them from a waiting list. As a result, their waiting experience differs from those clients who are considered a priority group.

23. Please estimate how many non-priority referrals your program receive in a month:

24. Provide an estimate of the average length of time between 1st contact and assessment?

25. In the last five years, has this length:

- [ ] Increased
- [ ] Decreased
- [ ] Stayed The Same

First Point of Contact for Non-Priority Clients

26. What events may have contributed to this change (e.g. change in policy, increase in clients, etc.)

Assessment

27. Does your service provide assessments?

- ◯ Yes formally
- ◯ Yes informally
- ◯ No

28. Provide an estimate of the average length of time between assessment and treatment (in days):

29. Has this length of time:

- [ ] Increased
- [ ] Decreased
- [ ] Stayed The Same

Assessment

30. What events may have contributed to this change (e.g. change in policy, increase in clients, etc)

31. Are clients informed of how long they will wait?

- ◯ Yes
- ◯ No

Drop Out

Pre-treatment attrition may be effected by wait times.

32. What is the average percentage of client drop out before receiving assessment?

33. What is the average percentage of client drop out before receiving treatment?

34. Is follow up performed on those clients who drop out?

- ◯ Yes
- ◯ No

Drop Out

35. What are the typical factors behind drop out as determined by follow up?

Wait Times

36. Does your organization keep a waiting list for your services?

- ◯ Yes
- ◯ No

Wait Times

37. Would you consider the wait list at your service overwhelming?

- ◯ Yes
- ◯ No

38. As of today, what is an estimate of how many people are currently on the waiting list?

39. Please describe how wait list time is defined (e.g. between first contact and assessment, between assessment and treatment, between first contact and treatment?)

40. What factors influence the size of the wait list? (e.g. low staff size, high demand)

41. In the last five years, has the number of clients on a wait list:

- ☐ Increased
- ☐ Decreased
- ☐ Stayed The Same

Wait Times

42. If there has been a change, what do you attribute this change to:

43. Does your agency offer any service to clients while they are waiting for an assessment?

- ◯ Yes
- ◯ No
- ◯ N/A

Wait Times

44. If yes, please describe the service:

45. Does your agency offer any service to clients while they are waiting for treatment?

- [ ] Yes
- [ ] No
- [ ] N/A

46. If yes, please describe the service:

47. Is there a maximum number of people allowed to be on a wait list?

- ( ) Yes
- ( ) No

Wait Times

48. What is the limit? (number of clients)

49. Has your agency implemented any particular processes or strategies to reduce or eliminate the wait list?

- Yes
- No

Wait Times

50. Please describe these strategies:

51. Have these strategies been successful in reducing the amount of time clients wait for services?

- ○ Yes
- ○ No

52. Please describe any barriers that exist to reduce wait times

53. Are systems in place to determine whether clients are on waiting lists at different addiction services in your community or across the province?

- ☐ Yes
- ☐ No

54. Does your centre discourage clients waiting for treatment at more than one service?

- ☐ Yes
- ☐ No

55. Will clients be placed on multpile wait lists for different services within your agency?

- ○ Yes
- ○ No

Survey is Complete

Thank you for taking the time to fill out this survey.

56. Is there any additional information regarding wait times for your services that you would like to share?
